# Adversarial Auxiliary Weighted Subdomain Adaptation for Open-Set Deep Transfer Bridge Damage Diagnosis

**DOI:** 10.3390/s23042200

**Published:** 2023-02-15

**Authors:** Haitao Xiao, Limeng Dong, Wenjie Wang, Harutoshi Ogai

**Affiliations:** 1 School of Information and Communication Engineering, Xi’an Jiaotong University, No. 28, Xianning West Road, Xi’an 710049, China; 2 Graduate School of Information, Production and Systems, Waseda University, 2-7, Hibikino, Wakamatsu-ku, Kitakyushu 808-0135, Japan; 3 School of Electronics and Information, Northwestern Polytechnical University, 127 West Youyi Road, Xi’an 710072, China

**Keywords:** structural damage diagnosis, transfer learning, MCMK-WLMMD, deep learning, adversarial learning

## Abstract

Deep learning models have been widely used in data-driven bridge structural damage diagnosis methods in recent years. However, these methods require training and test datasets to satisfy the same distribution, which is difficult to satisfy in practice. Domain adaptation transfer learning is an efficient method to solve this problem. Most of the current domain adaptation methods focus on close-set scenarios with the same classes in the source and target domains. However, in practical applications, new damage caused by long-term degradation often makes the target and source domains dissimilar in the class space. For such challenging open-set scenarios, existing domain adaptation methods will be powerless. To effectively solve the above problems, an adversarial auxiliary weighted subdomain adaptation algorithm is proposed for open-set scenarios. Adversarial learning is introduced to proposed an adversarial auxiliary weighting scheme to reflect the similarity of target samples with source classes. It effectively distinguishes unknown damage from known states. This paper further proposes a multi-channel multi-kernel weighted local maximum mean discrepancy metric (MCMK-WLMMD) to capture the fine-grained transferable information for conditional distribution alignment (sub-domain alignment). Extensive experiments on transfer tasks between three bridges verify the effectiveness of the algorithm in open-set scenarios.

## 1. Introduction

With the rapid development of artificial intelligence technology, deep learning has been widely studied and applied in the field of bridge structural damage diagnosis. A large number of deep learning-based bridge damage diagnosis techniques [1,2,3,4,5,6,7,8,9,10,11] have achieved better results than traditional damage diagnosis techniques in many aspects. Therefore, the focus of bridge structure damage diagnosis technology is gradually transitioning from the study of signal processing technology to the study of data intelligence-driven methods.

### 1.1. Related Work

Traditional deep learning models have achieved good results when the training (source domain) and test (target domain) datasets obey the same distribution [12]. However, due to variable loads and natural environments, the acquired diagnostic data of different bridges are in different distribution models (i.e., there is distribution discrepancy between the source and target domains). Therefore, the damage diagnosis knowledge learned from the source domain is less effective in the target domain. To solve these problems, the transfer learning (TL) theory has been extensively researched and discussed in recent years. The purpose of transfer learning is to seek the invariance between different domains by narrowing the discrepancy between them, and the method tries to improve the generalization ability and robustness of the model by utilizing samples from the source (labeled samples) and target (unlabeled samples) domains [13,14]. TL is widely used in the fields of image recognition, speech recognition [15], and fault diagnosis [16,17,18].

Deep transfer learning frameworks based on domain adaptation methods expect to learn shared features from the source and target domains to transfer damage knowledge [19,20,21], which is well suited to diagnosis problems. Domain adaptation mainly adopts two learning schemes: adversarial learning and the minimization of the distribution discrepancy metric between domains. Inspired by generative adversarial networks, domain adaptation based on adversarial learning reduces the feature distribution discrepancy between source and target domains by adversarially training the feature extractor and domain classifier [22]. The adversarial domain adaptation methods proposed by Long et al. [23], Chen et al. [24], and Li et al. [25] utilized feature extractors trained by source domain data to extract target features. Then, the feature distribution was aligned by maximizing the loss of the domain classifier, i.e., training the domain classifier with features from the source and target domains. Another domain adaptation scheme is to align feature distribution by minimizing distribution discrepancy metrics, such as MMD, JDA, JMMD, and RTML, to achieve efficient knowledge transfer. Lu et al. [26] and Wen et al. [27] used maximum mean discrepancy (MMD) for domain adaptive training and established a corresponding feature transfer model. The domain adaptation approach proposed by Lu et al. [28] achieved transfer learning by aligning distribution in multiple layers by minimizing MMD. To improve the performance of domain adaptation methods, Yang et al. [29] utilized a polynomial kernel to improve MMD, while Cao et al. [30] proposed a pseudo-classification to improve MMD for aligning inter-class distributions. Zhu et al. [31] and Che [32] used multi-kernel MMD to obtain good distribution alignment. Han et al. [33] and Qian et al. [34] used joint distribution adaptation (JDA) with pseudo-labels to align conditional and marginal distribution to construct a more efficient and robust feature representation for substantial distribution discrepancy. To reduce the marginal and conditional distribution discrepancy, Cao et al. [35] constructed an auxiliary soft label for joint MMD (JMMD) to enhance the performance of JMMD. Ding et al. [36] proposed a robust transfer metric learning method (RTML) framework that eliminates the difference between the boundary distribution and conditional distribution of the two domains in the sample space.

The application of TL in structural health monitoring (SHM) is an emerging field. The use of TL to solve the classification problem of vision-based SHM is becoming a new research direction [37]. In road crack detection, TL has proved to be an effective method for improving the accuracy of classification problems [38,39,40].

**Figure 1:**
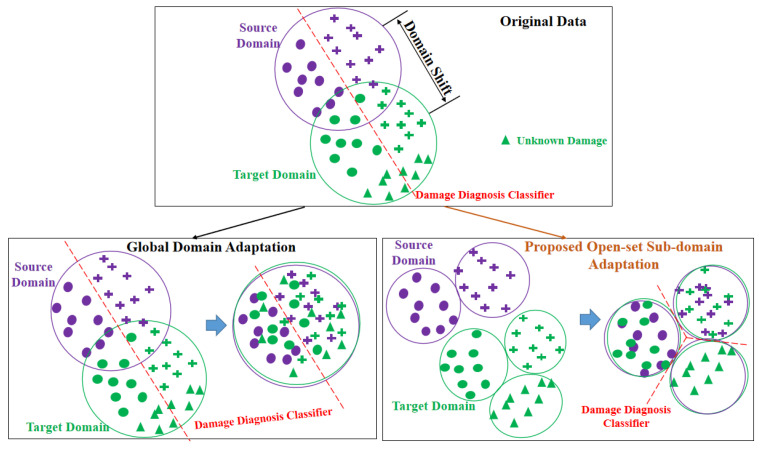
Domain shift and open-set domain adaptation methods.

From the discussion of the existing studies mentioned above, we know the following:

(1) The existing research has achieved good results in the close-set scenario; i.e., the source and the target domains have the same class space. However, in the actual bridge diagnosis scenario, new damages (unknown class) that are not included in the source domain classes often appear due to the degradation of the bridge structure; i.e., the class space of the source domain is a subset of the target domain (open-set) [41,42]. There are very few studies on fault or damage transfer diagnosis in such open-set scenarios. This brings a more challenging open-set domain adaptation problem to bridge damage transfer diagnosis, as shown in Figure 1. Figure 1 shows that the appearance of target outlier classes (new damage classes) brings the negative transfer of diagnostic knowledge to existing domain adaptation methods. This leads to a decline in the generalization ability of the model in the open-set scenarios [43].

(2) In the existing intelligent diagnosis methods based on TL, the domain adaptation method mainly learns the global domain shift to align the marginal distribution of the source and target domains without considering the relationship between the corresponding sub-domains (a sub-domain contains the samples within the same class). This confuses the data and the discrimination structure. As a result, fine-grained information of each class may be lost [44]. Figure 1 (left) shows an intuitive example that explains the confusion in the global domain adaptation. The data in different subdomains are too close to enable accurate classification. This is a common adaptation problem in the global domain.

### 1.2. Contributions

Motivated by the aforementioned issues and to promote the successful application of intelligent bridge damage diagnosis in open-set scenarios, this paper proposes a new intelligent structural damage diagnosis method for bridges, namely, an intelligent bridge diagnosis method based on adversarial auxiliary weighted subdomain adaptation network (AWSDN). A multi-channel multi-scale feature extractor is designed to expand the width of the feature extraction network to obtain deeper and multi-scale features. To isolate target outlier samples and prevent the negative transfer caused by these outliers, adversarial learning is introduced to the proposed adversarial auxiliary weights for samples in the target domain to describe the similarity between samples in the target and source domains. Furthermore, the multi-channel multi-kernel weighted local maximum mean discrepancy (MCMK-WLMMD) is proposed to effectively align the conditional distribution between correlated subdomains, i.e., subdomain adaptation. The main contributions of this paper are summarized as follows:

(1) Effectively solving the challenging open-set domain adaptation problem in bridge damage diagnosis, which is rarely studied in the existing literature.

(2) An adversarial weighting method is proposed for target samples by using adversarial training on the domain classifier and feature extractor. Negative transfer is avoided by isolating outlier class values with the help of adversarial auxiliary weights.

(3) MCMK-WLMMD aims to measure the distribution discrepancy between correlated subdomains in a shared class space to obtain fine-grained transferable information for more efficient domain adaptation.

(4) Extensive experiments on the dataset of three bridges verify the effectiveness and superiority of the proposed method.

This paper is organized as follows. The problem of TL is introduced in Section 2. In Section 3, the detailed designs of our proposed method are presented. In Section 4, field experiments and our analysis are discussed. The results prove that the proposed method is reliable, effective, and useful.

## 2. Preliminaries

### 2.1. Problem Formulate

This study focuses on the problem of open-set domain adaptation in bridge damage diagnosis. Usually, the labeled data obtained from a bridge or model are used as the source domain $D_{s}=\left\{\left(x_{i}^{s},y_{i}^{s}\right)\right\}$, where $n_{s}$ is the number of samples in the source domain, $x_{i}^{s}$ is the *i*th sample of the source domain, and $y_{i}^{s}$ is the label of the *i*th sample in the source domain ($y_{i}^{s}\in \left\{0,1,2,...,C-1\right\}$, where *C* is the number of sample labels). Accordingly, unlabeled data obtained from other working conditions or bridges are called the target domain $D_{t}=\left\{\left(x_{j}^{t}\right)\right\}$, where $n_{t}$ is the number of samples in the target domain, and $x_{j}^{t}$ is the *j*th sample of the target domain. The source domain data are collected under the probability distribution $P_{s}$, and the target domain data are collected under the probability distribution $P_{t}$, and $P_{s}\neq P_{t}$. In open-set domain adaptation, the label space $Y_{s}$ of the source domain is included in the class space $Y_{t}$ of the target domain. These classes of $Y_{s}$ are also called “shared classes”, i.e., $Y_{s}\subseteq Y_{t}$. Therefore, $P_{s}\neq P_{t,sh}$, where $P_{t,sh}$ represents the distribution of the target domain belonging to the source class space.

This paper aims to build a data-driven deep transfer model that can learn invariant features from source and target domains for bridge damage diagnosis in open-set scenarios. The deep transfer model can not only identify the target outliers as unknown classes but also accurately classify the target samples belonging to the shared classes.

### 2.2. Maximum Mean Discrepancy (MMD)

A parameter-free discrepancy metric called MMD is often used in many transfer tasks. It can estimate the distribution discrepancy between the different domains. The mathematical formula is as follows:

(1) $$\begin{array}{c} {\mathrm{MMD}}_{H}\left(X^{s},X^{t}\right)={∥\frac{1}{n_{s}}\sum_{i=0}^{n_{s}-1}\Phi \left(x_{i}^{s}\right)-\frac{1}{n_{t}}\sum_{j=0}^{n_{t}-1}\Phi \left(x_{j}^{t}\right)∥}_{H}^{2} \end{array}$$

where $X^{s}$ and $X^{t}$ are the sample sets of the source and target domains, respectively, and ${\mathrm{MMD}}_{H}\left(X^{s},X^{t}\right)$ is the distance between the source and target domain samples in the regenerated kernel Hilbert space. $H_{.}\Phi \left(\right)$ is the feature space mapping function. $n_{s}$ and $n_{t}$ are the number of samples in the source and target domains, and ${∥.∥}_{H}$ is a reproducing kernel Hilbert space. Minimizing Equation (1) can make the source and target domains closer, so that the model can more accurately predict the label of the sample in the target domain.

### 2.3. Convolutional Neural Network (CNN)

A CNN is a multi-layer feed-forward neural network that extracts features layer by layer through the alternation of connections of convolutional layers and pooling layers. A typical CNN generally uses a fully connected layer at the end of the network to integrate local information with category discrimination. Finally, a classifier such as Softmax is used for classification. The final loss function of the status recognition module based on the CNN is

(2) $$\begin{array}{c} Loss_{SR}\left(y,X\right)=\frac{1}{n_{s}}\sum_{i=0}^{n_{s}-1}J\left(y_{i}^{s},f\left(x_{i}^{s}\right)\right),\\ J\left(G,Q\right)=-\sum_{c=0}^{C-1}G_{c}lg\left(Q_{c}\right) \end{array}$$

where $f(x_{i}^{s})$ is the prediction result of the CNN with MPME for the *i*th sample of the source domain; J(), *G*, and *Q* are the cross-entropy loss function, one-hot encoding of the real label, and probability vector of the predicted label, respectively; and $G_{c}$ is a 0∼1 variable. When *c* is the true label of the sample, $G_{c}$ is 1; otherwise, it is 0.

## 3. Proposed Method

In this study, the original vibration signal of the bridge was used as the input for the intelligent structural damage diagnosis method. Using TL, the proposed method can achieve satisfactory diagnostic accuracy. The framework and training processes of our proposed intelligent structural damage diagnosis method are detailed in this section.

### 3.1. Sub-Domain Adaptation Deep Transfer Learning Network

**Figure 2:**
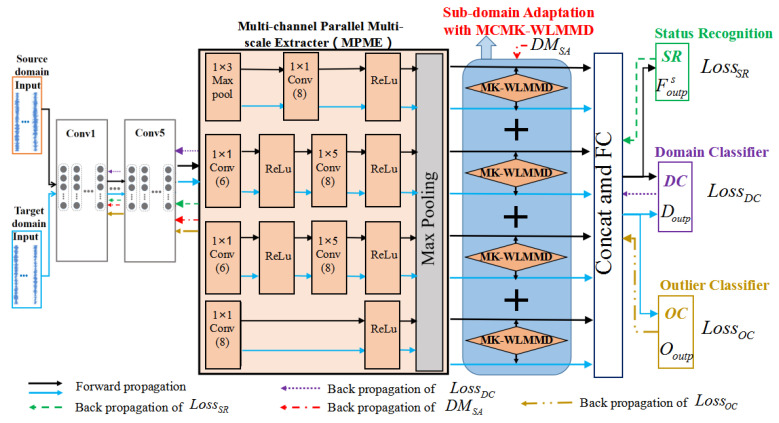
Framework of AWSDN.

In this section, a new deep learning framework, named AWSDN, is proposed for transfer damage diagnosis. The framework consists of four parts, as shown in Figure 2.

(1) Status Recognition Module (SR): This includes a feature extractor and a state recognizer. The feature extractor consists of a CNN and a multi-channel parallel multi-scale extractor (MPME) to automatically learn higher-level multi-scale features from input samples in different domains. The health status is determined by the status recognition based on the features extracted by the extractor.

(2) Adversarial Auxiliary Domain Classifier (DC): This takes the features learned by the feature extractor as input and predicts the domain labels of the features. Adversarial learning is introduced to use adversarial training between the domain classifier and feature extractor, meaning that the domain classifier cannot distinguish the domain classes of the features. Then, the prediction error of this domain classifier is used to describe the similarity of the target-domain samples with the source domain, i.e., the indicator of the adversarial auxiliary weight. Using this auxiliary weight indicator, the outlier samples (unknown class samples) of the target domain can be separated.

(3) Sub-Domain Adaptation Module with MCMK-WLMMD (SA): A sub-domain adaptation with multi-channel multi-kernel weighted local maximum mean discrepancy (MCMK-WLMMD) is proposed to align the conditional distribution for diagnosis acknowledgement transfer.

(4) Outlier Classifier (OC): This is used to identify the outlier classes (unknown classes) and shared classes of the target domain.

#### 3.1.1. Status Recognition Module (SR)

The vibration signal of the bridge in this study is a one-dimensional signal, and thus a 1D-CNN was selected. The 1D-CNN model used in status recognition is mainly composed of one input layer, five convolutional layers, five pooling layers, two fully connected layers, and one output layer. In the status recognition module, the first 13 layers are feature extractors, and the last layer is the health status classifier. The parameters of CNN and MPME are presented in Table 1 and Figure 2, where the original vibration signal data with length L are used as the input layer. *m*, *n*, and L are set as 5, 2, and 256, respectively.

#### 3.1.2. Adversarial Learning Based Target Instance Weighting

For unlabeled target domains, this paper proposes a weighting scheme assisted by adversarial learning. $W_{k}$ is set as the auxiliary weight for the *k*th target-domain sample, which represents the likelihood that the sample belongs to the shared class. This paper calculates the weights through two strategies, namely adversarial auxiliary weighting and inter-domain distance metric weighting.

A. Adversarial Auxiliary Weighting.

Figure 2 shows that the domain classifier is composed of a fully connected layer and an output layer, i.e., $FC_{DC}$ (two neurons) and $D_{output}$ (SoftMax). Based on the theory of domain learning discussed in [14] and Equation (2), the loss functions between the predicted domain label and the ground truth in domain classifier for the *i*th sample of source and target domains are expressed in Equation (3). Thus, the domain classifier loss can be written as in Equation (4). In Equation (3), $D_{i}^{s}$ and $D_{i}^{t}$ are the labels in the source and target domains, respectively. $f_{DC,i,k}^{s}$ and $f_{DC,i,k}^{t}$ represent the *k*th elements of $FC_{DC}$’s output for the source and target domains, respectively.

$$\begin{array}{c} Loss_{DC,i}^{s}=-\sum_{k=1}^{2}1\left\{D_{i}^{s}=k\right\}log\left(\frac{e^{f_{DC,i,k}^{s}}}{\sum_{m=1}^{2}e^{f_{DC,i,m}^{s}}}\right) \end{array}$$

(3) $$\begin{array}{c} Loss_{DC,i}^{t}=-\sum_{k=1}^{2}1\left\{D_{i}^{t}=k\right\}log\left(\frac{e^{f_{DC,i,k}^{t}}}{\sum_{m=1}^{2}e^{f_{DC,i,m}^{t}}}\right) \end{array}$$

(4) $$\begin{array}{c} Loss_{DC}=\frac{1}{n_{s}}\sum_{i=0}^{n_{s}-1}Loss_{DC,i}^{s}+\frac{1}{n_{t}}\sum_{i=0}^{n_{t}-1}Loss_{DC,i}^{t} \end{array}$$

In this study, the purpose of adding a domain classifier is to make the model unable to identify the domain label by maximizing the domain classifier loss. This means that it is difficult for domain classifiers to separate shared classes, while it is easy to separate outliers, through adversarial learning. Therefore, the domain prediction error of the domain classifier can be used as a similarity metric for the adversarial auxiliary weighting scheme. In this way, outlier samples of the target domain have smaller domain prediction errors, resulting in smaller adversarial auxiliary weights. From Equation (3), the adversarial auxiliary similarity weight $W_{A,i}$ of the *i*th target-domain sample can be defined as

(5) $$\begin{array}{c} W_{A,i}=Loss_{DC,i}^{t} \end{array}$$

B. Inter-Domain Distance Metric Weighting.

Although there is a domain gap between the source and target domains, the same classes in these domains generally have similar characteristics. Therefore, they should be closer in the high-level space. The centroids of the source-domain classes in the representation layer can be expressed as Equation (6).

(6) $$\begin{array}{c} C_{J}^{S}=\frac{1}{n_{j}^{s}}\sum_{x_{i}^{s}\in D_{j}^{s}}^{}f\left(x_{i}^{s}\right) \end{array}$$

where $G_{j}^{s}$ and $n_{j}^{s}$ represent the *j*th class of the source domain and the number of samples in the *j*th class, respectively. For the *k*th target sample, all distances between it and the source centroids are calculated, i.e., $l_{1},l_{2},...,l_{k},k=n_{G}$. Generally, when $l_{k}$ is smaller, the target sample is closer to the centroids of the source domain; i.e, the class of the target sample has a higher probability of belonging to the source domain. Therefore, the reciprocal of the minimum distance is selected as the weight $W_{L,k}$, as shown in Equation (7).

(7) $$\begin{array}{cc} W_{L,i} & =\frac{1}{l_{k,min}}\\ l_{k,min} & =\min_{j=1,2,...,n_{G}}∥f\left(x_{k}^{t}\right)-c_{j}^{s}∥ \end{array}$$

C. Joint Target Instance Weighting.

In this study, the proposed target instance weight combines the adversarial auxiliary weight with the inter-domain distance metric weight, i.e., the joint target instance weight expressed as Equation (8). In addition, to obtain normalized weights, the weights need to be properly scaled by normalization. The min–max normalization shown in Equation (9) is used to normalize these two weights. After normalization, Equation (8) can be rewritten as Equation (10). After normalizing the joint weights, they are attached to the target samples for subdomain adaptation. In this way, in MCMK-WLMMD-based subdomain adaptation, the distribution between target and source domains in shared classes is aligned, while target outliers are isolated.

(8) $$\begin{array}{cc} W_{i} & =W_{A,i}+W_{A,i}\\ {\hat{W}}_{A,k} & =\tau \frac{W_{A,k}-W_{A,\min}}{W_{A,\max}-W_{A,\min}+\omega } \end{array}$$

(9) $$\begin{array}{cc} {\hat{W}}_{L,k} & =\tau \frac{W_{L,k}-W_{L,\min}}{W_{L,\max}-W_{L,\min}+\omega }\\ W_{A,\max} & =\max\left(W_{A,k}\right),W_{A,\min}=\min\left(W_{A,k}\right)\\ W_{L,\max} & =\max\left(W_{L,k}\right),W_{L,\min}=\min\left(W_{L,k}\right) \end{array}$$

(10) $$\begin{array}{cc} W_{K} & ={\hat{W}}_{A,k}+{\hat{W}}_{L,k} \end{array}$$

#### 3.1.3. Sub-Domain Adaptation with MCMK-WLMMD

Most of the studies using domain adaptation for TL [42,43,44,45] focus on using MMD to reduce the edge distribution discrepancy (global distribution) between the target and source domains without considering the relationship between the two sub-domains in the source and target domains. Thus, the conditional distribution discrepancy between the source and target domains is ignored. This is a common problem in the previous global domains.

A. Weighted Local Maximum Mean Difference (WLMMD).

To solve the above problems, the local distribution of the two sub-domains in the source and target domains is used in this study. By minimizing the local distribution discrepancy between the two sub-domains of the source and target domains, sub-domain adaptation (also known as aligning condition distribution) is achieved. However, in unsupervised TL, the samples of the target domain are unlabeled, which makes it difficult to use the MMD to align the conditional distribution between the source and target domains. In response to this situation, the WLMMD is proposed to achieve proper alignment. WLMMD uses the output of the training network $y=f(x^{t})$ as the pseudo-label of the target domain samples. For the target domain, the pseudo label may be incorrect, and using this incorrect label will reduce the performance. Therefore, a probabilistic prediction (soft prediction) is proposed to mitigate this negative impact. The WLMMD measures the distribution discrepancy of related sub-domains in the source and target domains while considering different sample weights. According to weight $\omega_{c}$, each sample should belong to a class. Therefore, based on this, Equation (11) is obtained. By minimizing Equation (11), the distribution of the sub-domains in the same class can be closer.

(11) $$\begin{array}{cc} {\hat{d}}_{H} & =\frac{1}{C}\sum_{c=0}^{C-1}{∥\sum_{i=0}^{n_{s}-1}\omega_{i,c}^{s}\Phi \left(x_{i}^{s}\right)-\sum_{j=0}^{n_{s}-1}\omega_{i,c}^{t}\Phi \left(x_{j}^{t}\right)∥}_{H}^{2},\\ & where\;\;\omega_{i,c}=\frac{y_{i,c}}{\sum_{j=0}^{n-1}y_{i,c}} \end{array}$$

where ${\hat{d}}_{H}$ is the distribution discrepancy measurement considering the effect of pseudo-labels with probabilistic prediction. $\omega_{i,c}^{s}$ and $\omega_{j,c}^{t}$ represent the weights of $x_{i}^{s}$ and $x_{j}^{t}$ belonging to category *c*, respectively. Note that both $\sum_{i=0}^{n_{s,c}-1}\omega_{i,c}^{s}$ and $\sum_{j=0}^{n_{s,c}-1}\omega_{j,c}^{s}$ are equal to 1, and $\sum_{i=0}^{n_{c}-1}\omega_{i,c}$ is the weighted sum of category *c*. $y_{i,c}$ is the $c^{th}$ item of the vector $y_{i}$. For the samples in the source domain, it uses the real label $y_{i}^{s}$ as the one-hot vector to calculate the $\omega_{i,c}^{s}$ of each sample. However, in unsupervised adaptation, the target domain is unlabeled data, and it cannot directly use $y_{j}^{t}$ to calculate $\omega_{j,c}^{t}$. Thus, the output of the training network $\hat{y}=f\left(\right)$ is used as a pseudo-label of the target domain samples to calculate the $\omega_{j,c}^{t}$ of each sample in the target domain. Then, the $\omega_{j,c}^{t}$ can be calculated for each target sample. Finally, Equation (11) is calculated.

B. MCMK-WLMMD Alignment.

**Figure 3:**
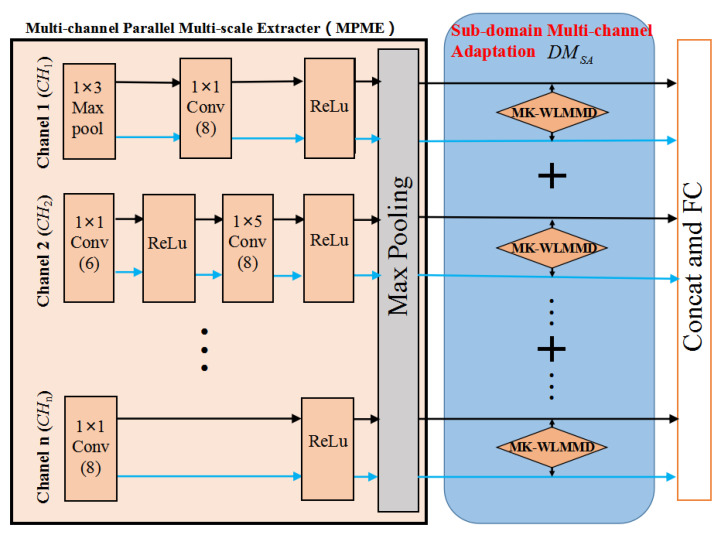
Framework of sub-domain adaptation with MCMK-WLMMD.

Because the high-order features directly affect the capability of damage transfer, the adaptation alignment of the sub-domain distribution is realized by reducing the distribution discrepancy between the relevant sub-domain distributions in these channels ($CH_{1}$, $CH_{2}$, and $CH_{n}$), as shown in Figure 3.

First, the hidden representations of $CH_{1}$, $CH_{2}$, and $CH_{n}$ are mapped to reproducing kernel Hilbert spaces (RKHSs), and the source and target domains outputs are set as $f_{CH_{1}}^{s}$ and $f_{CH_{1}}^{t}$, $f_{CH_{2}}^{s}$ and $f_{CH_{2}}^{t}$, and $f_{CH_{n}}^{s}$ and $f_{CH_{n}}^{t}$, respectively.

Second, in the RKHS space, to avoid the fact that a single kernel function is not conducive to a proper expression of the mapping relationship between the two spaces, this study selects multiple Gauss kernel (MK) functions to enhance the representation ability of the mapping function. The multi-kernel function (MK) is given by Equation (12). Therefore, based on Equation (12), it can obtain the MK-WLMMD in $CH_{1}$, $CH_{2}$, and $CH_{n}$ by using Equation (13), where $z=(1,2,...,n_{CH})$ ($n_{CH}=4$ in this study), and *C* is the number of categories. By minimizing Equation (14), the condition distribution between the source and target domains is aligned to realize sub-domain adaptation. Therefore, the error of sub-domain adaptation with MCMK-WLMMD can be expressed as Equation (14).

(12) $$\begin{array}{cc} \kappa ≜\{K & =\sum_{\mu =0}^{m-1}\beta_{\mu }k_{\mu }:\beta_{\mu }\geq 0,\forall \mu \} \end{array}$$

(13) $$\begin{array}{cc} \hat{d}\left(f_{CH_{z}}^{s},f_{CH_{z}}^{t}\right) & =\frac{1}{C}\sum_{c=0}^{C-1}[\sum_{i=0}^{n_{s,c}-1}\sum_{j=0}^{n_{s,c}-1}\omega_{i,c}^{s}\omega_{j,c}^{s}\kappa \left(f_{CH_{z},i}^{s},f_{CH_{z},j}^{s}\right)\\ & +\sum_{i=0}^{n_{t,c}-1}\sum_{j=0}^{n_{t,c}-1}\omega_{i,c}^{t}\omega_{j,c}^{t}\kappa \left(f_{CH_{z},i}^{s},f_{CH_{z},j}^{t}\right)\\ & -2\sum_{i=0}^{n_{s,c}-1}\sum_{j=0}^{n_{s,c}-1}\omega_{i,c}^{s}\omega_{j,c}^{t}\kappa \left(f_{CH_{z},i}^{s},f_{CH_{z},j}^{t}\right)] \end{array}$$

(14) $$\begin{array}{c} DM_{SA}=\sum_{z=1}^{n_{CH}}\hat{d}\left(f_{CH_{z}}^{s},f_{CH_{z}}^{t}\right) \end{array}$$

#### 3.1.4. Outlier Classifier

**Figure 4:**
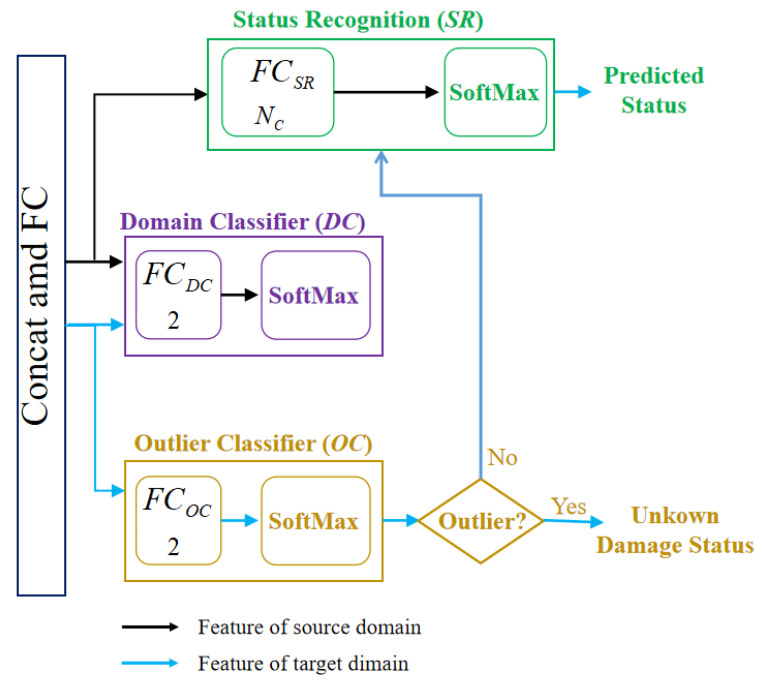
Classifiers of AWSDN.

As shown in Figure 4, the model contains three classifiers: the state recognition (SR), the domain classifier (DC), and the outlier classifier (OC). The state recognition is designed to identify the bridge health states under source supervision, and its loss function is shown in Equation (2). The purpose of the domain classifier is to discriminate whether the samples belong to the source or target domains. This is a two-class classification problem based on supervision. The cross-entropy loss function of DC is shown in Equation (4).

The outlier classifier OC is designed to accurately identify outlier states in the target domain. Since the samples in the target domain are unlabeled data, this study proposes pseudo-outlier labels for the target samples to train the outlier classifier. As described in Section 3.1.2, the loss function of DC can be used as an outlier indicator to assist in labeling target-domain samples. Object samples from the shared classes are often hard to distinguish, which leads to larger errors. At the same time, the target outliers are more different from the source domain, resulting in a smaller prediction error. Therefore, for the normalized similarity weight of the target domain, i.e., $W_{k}$, the samples larger than the threshold are considered as shared classes, and those below the threshold are outliers, i.e., unknown classes. In this way, pseudo outlier labels can be appended to unlabeled target samples to train the outlier classifier OC. The loss function of DC for the $i^{t}h$ target sample can be expressed as Equation (15), where denotes the pseudo outlier label. Therefore, the loss function of the outlier classifier can be written as Equation (16). When the feature of the target domain is classified as an outlier state by OC, the damage state of this target domain sample is identified as the unknown damage state (new damage). When OC identifies the feature of the target domain as a known state (shared class), this feature is fed into the SR to further identify the detailed damage state (labeled shared class).

(15) $$\begin{array}{c} Loss_{OC,i}^{t}=-\sum_{k=1}^{2}1\left\{O_{i}^{t}=k\right\}log\left(\frac{e^{f_{OC,i,k}^{t}}}{\sum_{m=1}^{2}e^{f_{OC,i,m}^{t}}}\right) \end{array}$$

(16) $$\begin{array}{c} Loss_{OC}=\frac{1}{n_{ps}}\sum_{i=0}^{n_{ps}-1}Loss_{OC,i}^{s} \end{array}$$

### 3.2. Optimization Objective

The training optimization objective of the AWSDN mentioned in this study consists of four parts:

(1) Minimizing the status recognition error in the source domain;
(2) Maximizing the error of the domain classifier;
(3) Minimizing the error of the sub-domain adaptation with MCMK-WLMMD;
(4) Minimizing the error of the outlier classifier.

#### 3.2.1. First Objective

In the status recognition module, because there are sufficient damage state labeled data in the source domain, the training of the AWSDN model on the source domain adopts the supervised learning mode. After the model completes the feature extraction, the status recognition loss ($Loss_{SR}$) between the predicted class (predicted by the model) and the actual class of the source domain is calculated. The calculation formula is the same as Equation (2). Then, the first optimization objective is expressed in Equation (17).

(17) $$\begin{array}{c} \min_{f}\frac{1}{n_{s}}\sum_{i=0}^{n_{s}-1}J\left(y_{i}^{s},f\left(x_{i}^{s}\right)\right) \end{array}$$

#### 3.2.2. Second Objective

As shown in Figure 2, the domain classifier was designed to learn domain invariant features; that is, the domain classifier cannot distinguish the features between the source and target domains. Therefore, the second object is to maximize the domain classification error (i.e., $Loss_{DC}$), i.e., Equation (18).

(18) $$\begin{array}{c} \max_{f}\left(\frac{1}{n_{s}}\sum_{i=0}^{n_{s}-1}Loss_{DC,I}^{S}+\frac{1}{n_{t}}\sum_{j=0}^{n_{t}-1}Loss_{DC,j}^{t}\right) \end{array}$$

#### 3.2.3. Third Objective

The sub-domain adaptation with MCMK-WLMMD is proposed to reduce the condition distribution discrepancy between the source and target domains, i.e., minimizing the error of sub-domain adaptation ($D_{SA}$). Therefore, the third objective is shown as Equation (20).

(19) $$\begin{array}{c} \min_{f}\sum_{z=1}^{n_{CH}}\hat{d}\left(f_{CH_{z}}^{s},f_{CH_{z}}^{t}\right) \end{array}$$

#### 3.2.4. Fourth Objective

Outlier classifier is designed to recognize the new damage in the target domain; thus, the fourth objective is to minimize the error of the outlier classifier, i.e., Equation (20).

(20) $$\begin{array}{c} \min_{f}\frac{1}{n_{ps}}\sum_{i=1}^{n_{ps}}Loss_{OC,i}^{t} \end{array}$$

### 3.3. Optimization Objective Training

Based on the above information on optimization, the overall optimization objective is to maximize the error of the domain classifier under the premise that the sum of the errors of the status recognition with sub-domain adaptation and outlier classifier in their respective domains is minimized. Thus, the final object is to minimize Equation (21).

(21) $$\begin{array}{c} Loss_{total}=Loss_{SR}+\lambda DM_{SA}+\eta Loss_{oc}-\gamma Loss_{DC} \end{array}$$

After establishing the training optimization object, AWSDN can use the fast gradient descent algorithm (SGD) to train the proposed method. To complete the training process by minimizing $Loss_{tatal}$, the trained network (AWSDN) can learn domain-invariant features, so that the trained network can recognize the new damage status and accurately predict the labels of the samples in the target domain. Therefore, the AWSDN can be used to transfer damage diagnosis between different bridges and working conditions in open-set.

## 4. Field Bridge Experiment Result Study

**Figure 5:**
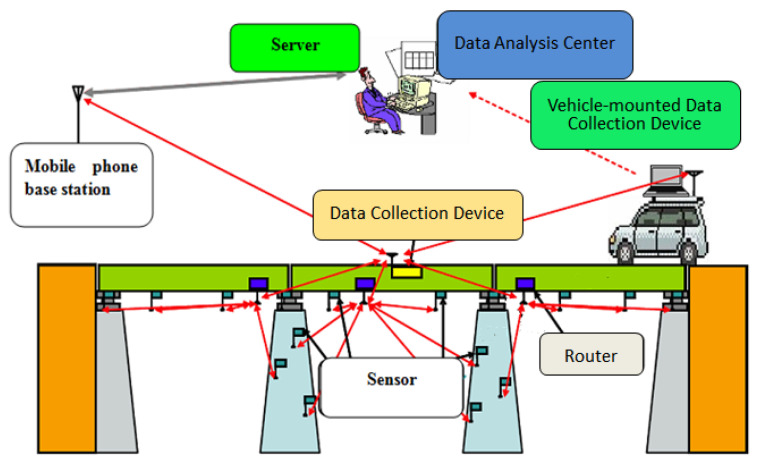
Sketch of IBSHM.

From 2008 to 2019, an intelligent bridge structural health monitoring system (IBSHM) was developed to gather vibration data and evaluate the healthy condition automatically and in real time. A schematic of this system is shown in Figure 5. To improve the IBSHM, it was tested on 10 bridges in Japan using a variety of experiments.

To promote the successful application of intelligent damage diagnosis of bridges with unlabeled data, this paper introduces experiments on the Kando Bridge, the simulation model of the Kando Bridge, and the Seiran Bridge to verify the reliability, superiority, and transfer ability of AWSDN. We used three data sets obtained from three different bridges (a test bridge named Kando Bridge, Kando Bridge’s simulation model, and a similar structure bridge named Seiran Bridge) to perform transfer damage diagnosis experiments and data analysis.

### 4.1. Data Set

**Figure 6:**
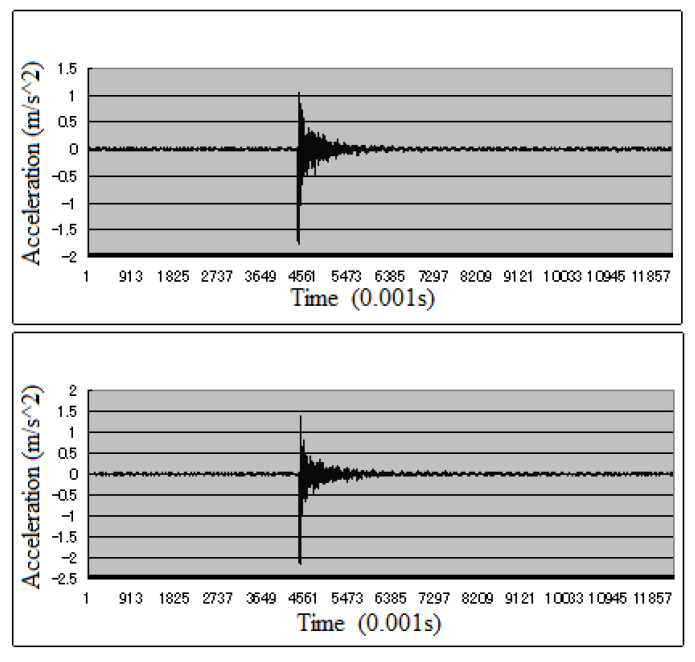
Acceleration data of one sensor in Kando Bridge.

The data-sets of these three bridges (data-set A, data-set B, and data-set C were acquired from Kando Bridge, Kando Bridge’s simulation model, and Seiran Bridge, respectively) were established to carry out the research on deep TL damage diagnosis. Thus, the three datasets were distributed in three different domains, and the data distribution in each domain was different. In these experiments, acceleration sensors with 200 Hz sampling frequency were deployed on bridges. For example, in the Kando Bridge experiment, 300 iterations of data collection by 15 wireless sensors and 300 iterations of data collection by 15 wired sensors were performed for each damage and excitation situation. In total, 400 samples was selected randomly from these 600 samples for data analysis. The acceleration data of one sensor (in the case 1 of damage type III) is shown in Figure 6 The upper picture shows the data of the intact bridge, and the lower picture shows the data after damage. Therefore, each dataset has 2800 samples, including seven different bridge states (seven categories of labels, or seven sub-domains); i.e., the number of samples with the same label in each domain is 400. The information of these three bridges and their corresponding datasets is introduced in detail in the next three subsections.

#### 4.1.1. Data Set A

**Figure 7:**
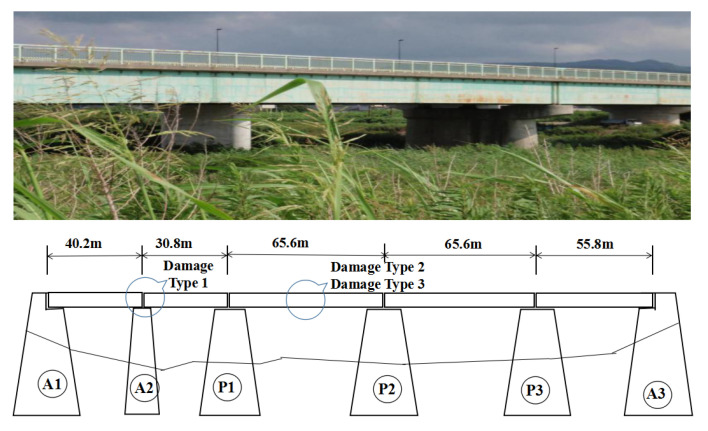
Kando Bridge.

Data-set A was obtained from a test bridge named Kando Bridge. The old Kando Bridge was built in 1964 in Izumo, Shimane Prefecture, Japan. Since the new Kando Bridge was built, the old bridge was abandoned and used for various damage diagnosis experiments. Experiments were conducted to acquire undamaged and damaged bridge data. The bridge is a steel girder bridge with a concrete deck slab. Figure 7 shows a schematic showing the location of the damage on the bridge.

Before we artificially damaged the bridge, we collected data to serve as the measurement of the undamaged condition (although these data may not reflect the actual bridge condition). To obtain significant vibration data in the damage position, we deployed sensors beside the potential damage in this experiment. We then induced damage to the bridge and collected measurement data, which were considered to represent the damage condition.

Damage was inflicted at three different locations in two cases, as listed in Table 2.

**Figure 8:**
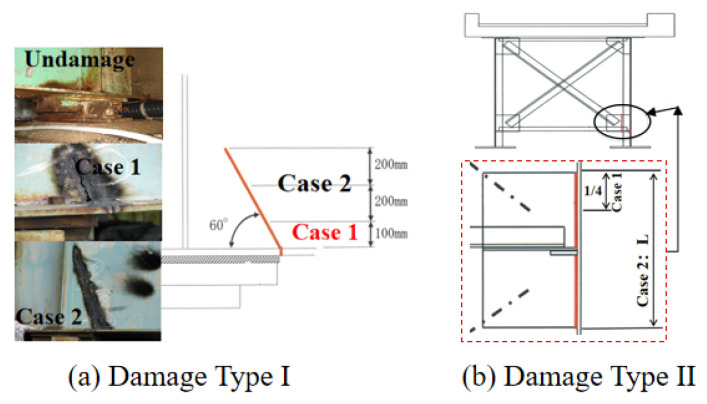
Damage type I and II.

**Figure 9:**
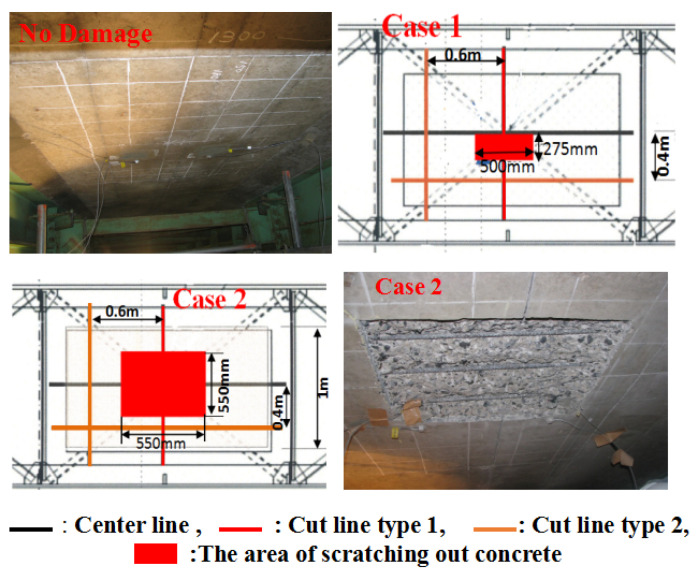
Damage type III.

The detailed information of these damage types is as follows:

(1) Type I: The bearing between the abutment and bridge deck was damaged, as shown in Figure 8a, cutting the auxiliary steel plate at 100 mm and 500 mm, respectively.

(2) Type II: The gusset plate of the bridge deck located between piers P1 and P2 was damaged, as shown in Figure 8b, and the horizontal gusset L was cut.

(3) Type III: The concrete ceiling and the reinforcement were scratched out to damage the deck of the bridge, as shown in Figure 9, where the cut depth of cut line type 1 is 25 mm. The depth of cut line type 2 in the horizontal direction was 25 mm, and the depth in the vertical direction was 3 mm. The depths of the area where concrete was scratched out in case 1 and case 2 were 10 mm and 30 mm, respectively (no damage to the rebars).

After measuring the non-damaged Kando Bridge, we performed the above three types of artificial damage to the bridge. Because the most common excitation in normal bridges is a running car, apart from ambient excitation, a loaded moving car was used as an external excitation. For damage type I, II, and III, the sampling frequency of the acceleration sensors was 200 Hz.

#### 4.1.2. Data Set B

**Figure 10:**
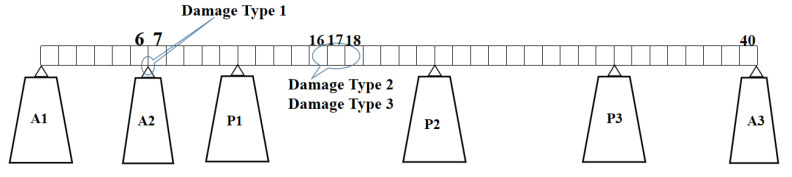
Kando Bridge model.

Dataset B was obtained from the simulation model of the Kando Bridge. The model is shown in Figure 10, where the length is 258 m. It is divided into 40 units (one sensor node per unit). The elastic modulus of the material is E = 2.06 × 108 kN/m${}^{2}$, Poisson’s ratio σ=0.3, and density ρ = 7850 kg/m${}^{3}$. The structural damage is mainly reflected in the decrease in stiffness. In this study, the reduction in the elastic modulus E of the material was used to simulate damage (indicated in Table 3), and stochastic white noise was used as excitation. ANSYS was used to establish the finite element model of a continuous beam bridge.

#### 4.1.3. Data Set C

**Figure 11:**
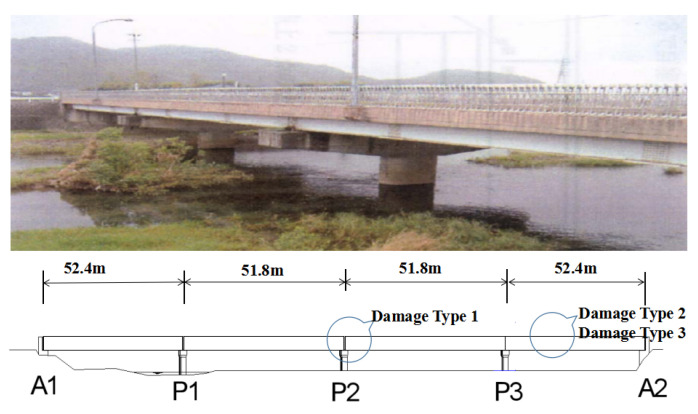
Seiran Bridge.

The experimental bridge was called Seiran Bridge. This bridge satisfied all the demand conditions that we required: made of steel, with deterioration problems, many crossing cars, and easy placement of the bridge sensor module. In other words, this bridge is typical of Japan’s bridge problems. Figure 11 shows the bridge and damage locations.

**Figure 12:**
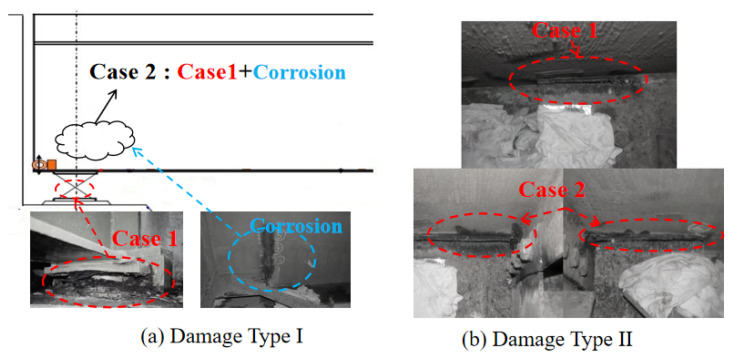
Damage type I and II of Seiran Bridge.

**Figure 13:**
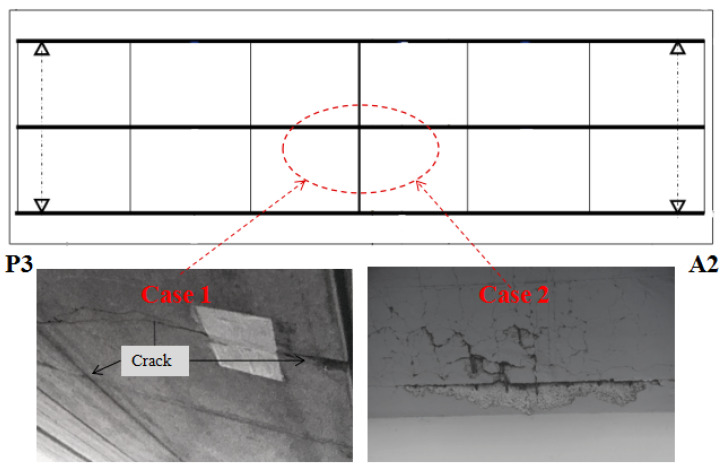
Damage type III of Seiran Bridge.

Damages include corrosion and deterioration at three different places, as indicated in Table 4. The detailed information of these damage types is as follows:

(1) Type I: The bearing between the abutment and bridge deck was corroded, as shown in Figure 12a.

(2) Type II: The steel frame of the bridge deck located between piers P1 and A2 was corroded, as shown in Figure 12b.

(3) Type III: The concrete ceiling of the deck was deteriorated as shown in Figure 13.

### 4.2. Pen-Set Transfer Tasks and Details

In this study, to evaluate the damage recognition performance of our proposed method in the open set scenarios, the source domain only covers part of the damage states in different transfer tasks. The open-set transfer tasks are listed in Table 5. Each open-set transfer task is represented by the symbol A→B, where A is the source domain data collected under Kando Bridge (dataset A), and B is the target domain data collected under Kando Bridge’s simulation model (dataset B). TL uses 2800 labeled samples in the source domain, 50% unlabeled samples (1400) in the target domain for training, and 1400 samples in the target domain for testing.

### 4.3. Methods for Comparison

To verify the effectiveness and superiority of the AWSDN in open-set scenarios, this study also uses other models to conduct a comparative analysis. The related models are presented in Table 6.

(1) CNN (M1): A deep learning method based on the same scheme as the CNN in AWSDN. It is a supervised learning method only considering the source domain as a loss function. Compared with M1, it aims to illustrate the improvement of the deep transfer damage diagnosis method.

(2) Open-set support vector machine (OSVM, M2) [45]: OSVM utilizes a probability threshold to detect outliers, and when the predicted probability is less than the threshold, the sample is identified as an outlier. This method utilizes supervised learning to train the network structure. Then, it focuses on the data representation learned by the trained network to implement OSVM. This comparison aims to illustrate the impact of the learned features on transfer learning.

(3) OSVM-MMD (M3): OSVM-MMD combines transfer learning techniques with OSVM. The transfer learning is achieved by minimizing the MMD metric between the source and target domains to improve the identification of OSVM.

(4) Deep TL (DDC, M4) [46]: To demonstrate the advantage of our proposed model, AWSDN is compared with the existing advanced and widely used deep TL method M4. Based on M1, M4 adds an adaptive layer based on the MMD and uses the learning features for domain adaptation. In the adaptive layer, a single Gaussian kernel was used to calculate the distribution distance MMD. The optimization goal is to minimize the MMD loss and the classification loss to reduce the marginal distribution discrepancy between the source and target domains for TL. The CNN model structures of M1 and M4 are presented in Table 1, where the convolution kernel is 5, the pooling kernel is 2, and the modified linear unit (ReLU) is mainly used as the activation function.

(5) DCTLN (M5) [47]: This is an adversarial domain adaptation method. DCTLN uses single-kernel MMD with adversarial learning for effective domain adaptation.

### 4.4. Result Analysis and Comparison

#### 4.4.1. Cross-Bridge Damage Diagnosis Result Analysis

**Figure 14:**
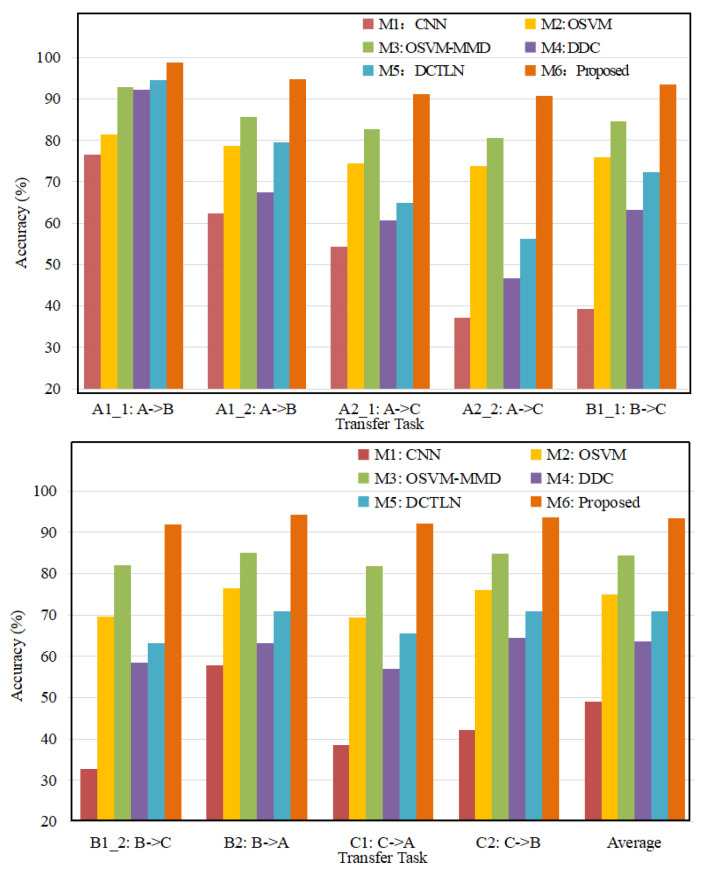
Diagnosis results of six methods in accuracy (%).

In this study, to evaluate performance, target outliers are treated as unknown classes. Figure 14 lists the transfer damage diagnosis accuracy of the six models, and the calculation formula is shown as Equation (22).

(22) $$\begin{array}{c} a=\frac{1}{n_{t}}\sum_{j=0}^{n_{t}-1}sign\left(f\left(x_{j}^{t}\right)=y_{j}^{t}\right) \end{array}$$

where sign() is the indicator function, $y_{j}^{t}$ is the true label of the *j*th sample in the target domain, and $f(x_{j}^{t})$ is the prediction result of the model on the *j*th sample in the target domain.

**Figure 15:**
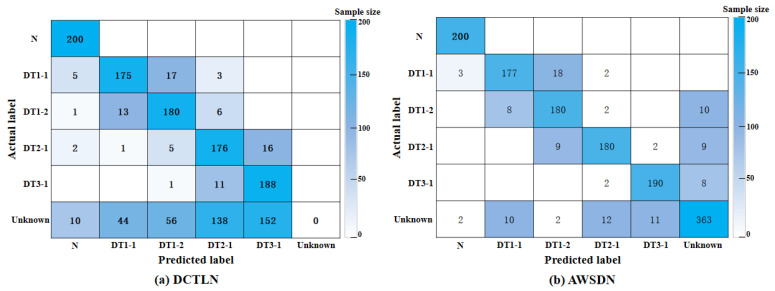
Confusion matrix of the diagnosis results in $C_{1}:C\to A$.

As can be observed in Figure 14, the proposed model, AWSDN, is superior to other models in open-set transfer tasks. Figure 15 shows the results further analyzed with the confusion matrix. These results confirmed the superiority of the model and allowed us to obtain the following conclusions.

(1) AWSDN achieved the best results for all open-set transfer tasks. The high accuracy rate highlights the superior generalization ability and robustness of AWSDN. It can effectively address the challenging open-set transfer damage diagnosis and provide more accurate diagnosis results.

(2) In the open-set damage diagnosis, because M1 (CNN) is trained only through source domain data and ignores the discrepancy in the distribution of data collected under different bridges or different environments, the results of M1 are far from ideal. In addition, the samples of the target outlier classes lead to a significant increase in the recognition error rate of M1. However, AWSDN reduces the distribution discrepancy and separates the samples of the target outlier classes by proposing an adversarial auxiliary weighted sub-domain adaptation module to obtain a better classification accuracy. Experimental results show that, compared with traditional CNN (M1), the classification accuracy of AWSDN is increased by 48%, which is significantly better than CNN. This means that TL can promote the successful application of the intelligent damage diagnosis of bridges in the case of open-set scenarios.

(3) Compared with the effective approach for outlier detection (OSVM), the diagnosis accuracy of the two methods with transfer learning and outlier detection (OSVM-MMD and AWSDN) is significantly better. The reason is that OSVM-MMD and AWSDN reduce the distribution discrepancy between the source and target domains through the domain adaptive method, while OSVN cannot well address the domain shift. By combining MMD-based domain adaptation with OSVM, OSVM-MMD not only extracts domain-invariant features, but also separates outliers, which greatly improves the diagnostic accuracy.

(4) The deep TL method DDC (M4) and DCTLN (M5) show better diagnostic performance than M1 because of the use of domain adaptation to reduce the domain distribution discrepancy. However, since DDC and DCTLN do not consider the negative transfer caused by outlier samples in the target domain, their diagnostic accuracy is greatly reduced. Therefore, the diagnosis results of these two deep transfer learning methods are significantly worse than AWSDN. Experimental results show that, compared with DDC (M4) and DCTLN (M5), the classification accuracy of AWSDN is increased by approximately 32% and 24%. This means that, compared with the widely used deep TL methods (such as DDC) and recent deep TL methods (DCTLN), AWSDN uses adversarial auxiliary weighting to isolate outliers to avoid negative migration caused by outliers and designs an outlier classifier to identify unknown classes. Otherwise, AWSDN uses the MCMK-WLMMD-based sub-domain adaptation to minimize the conditional distribution discrepancy enabling the convolutional network to better handle the open-set scenario.

(5) Figure 15 shows the confusion matrix of the diagnosis results of DCTLN and AWSDN for task $C_{1}:C\to A$. Table 7 lists the accuracy and recall rate of AWSDN in the transfer task $C_{1}:C\to A$. As can be observed in Figure 15 and Table 7, the health state of the bridge can be easily and correctly identified by AWSDN. The average precision and average recall rate of AWSDN were both above 94%. However, DCTLN cannot achieve good class-level alignment due to the interference of target outliers. Therefore, lower diagnostic accuracy is obtained in the open-set scenario. Figure 15 and Table 7 confirm the effectiveness and practicability of AWSDN on the other hand. The calculation formulas for the precision *P* and the recall rate *R* of label *c* are expressed in Equations (23) and (24) respectively.

(23) $$\begin{array}{c} P=\frac{n_{c}^{TP}}{n_{c}^{TP}+n_{c}^{FP}} \end{array}$$

(24) $$\begin{array}{c} R=\frac{n_{c}^{TP}}{n_{c}^{TP}+n_{c}^{FN}} \end{array}$$

where $n_{c}^{TP}$ is the number of samples whose true label and predicted label are both *c*. $n_{c}^{FP}$ is the number of samples whose predicted label is *c*, but the true label is not *c*. $n_{c}^{FN}$ is the number of samples whose true label is *c*, but the predicted label is not *c*.

#### 4.4.2. Visualization Analysis

**Figure 16:**
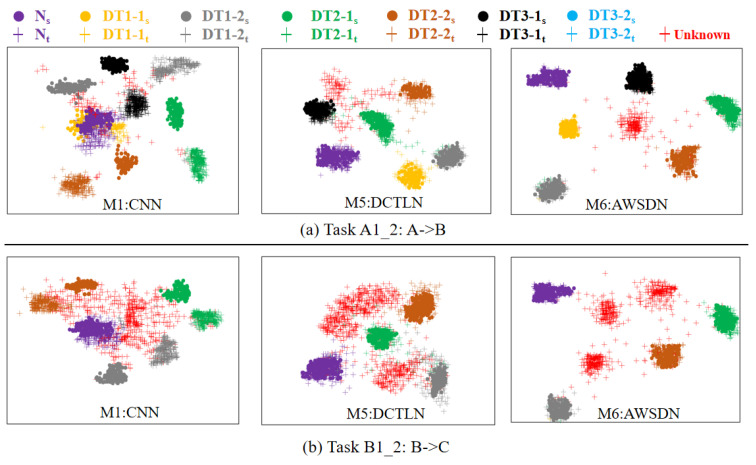
Feature visualization using *t*-SNE.

To reveal the ability of AWSDN to align the features of the same label samples (sub-domains) in different domains, Figure 16 shows the results of high-order feature visualization using *t*-SNE [40]. These features are the high-order features of the source domain and target domain samples processed by these methods in the transfer task $A_{1,2}:A\to B$ and $B_{1,2}:B\to C$. The additions of s and t in front of the status symbols represent the features of the source and target domains, respectively. The results of $A_{1,2}:A\to B$ shows that the visualized features of AWSDN have the smallest number of error clusters, and the samples of the same class in different domains are clustered in the same area. At the same time, AWSDN can properly isolate the target outlier classes in different regions. Therefore, the diagnosis knowledge learned from the source domain can be well transferred to the target domain, and the unknown classes in the target domain can also be accurately identified. This means that AWSDN can accurately align the distribution between the source and target domains in open-set scenarios, enabling sub-domain adaptation. However, the clustering results obtained by CNN show that the samples of the same label in different domains are far apart and overlap with the samples of other labels. This can easily lead to errors in model classification and a decrease in diagnostic accuracy. The clustering effect of DCTLN (M5) with the adversarial learning and MMD is significantly improved compared to that of CNN, but there is still a significant overlap between target outliers and known classes, which leads to a significant drop in the transfer diagnostic performance under the source domain supervision. The fundamental reason for this phenomenon is that DCTLN ignores the negative transfer caused by outliers and the conditional distribution discrepancy between domains. Thus, the use of adversarial auxiliary weighting and sub-domain adaptation with MCMK-WLMMD can further reduce the negative effects of outliers and distribution discrepancy. This finding indicates that the AWSDN model can divide samples more clearly than other models.

## 5. Conclusions

In this study, deep transfer learning was introduced for the first time in the field of bridge structural damage diagnosis to promote the successful application of intelligent bridge damage diagnosis in open-set scenarios. To solve the distribution discrepancy between domains in the open-set scenarios, an adversarial auxiliary weighted subdomain adaptation-based deep transfer learning model was proposed. A sub-domain adaptation module based on MCMK-WLMMD was proposed to obtain domain-invariant features. To prevent the negative transfer caused by the outliers, an adversarial auxiliary weighting mechanism was proposed to obtain the instance-level weights of the target-domain samples, which were used to describe the similarity of target-domain samples with the source. An experimental study of open-set deep transfer damage diagnosis was also conducted. From the results, the following conclusions were drawn:

(1) First, compared with the deep learning based intelligent structure damage diagnosis method without the transfer learning and outlier classes isolation, our proposed method has a higher recognition accuracy in open-set scenarios.

(2) Second, our proposed adversarial auxiliary weighted sub-domain adaptation with MCMK-WLMDD is superior to the domain adaptation of other widely used TL methods in terms of minimizing the distribution discrepancy between different domains.

(3) Finally, our proposed method can extend the network trained with labeled data obtained from one bridge to classify the unlabeled data with unknown classes in the open-set scenarios. This will promote the practical application of the transfer damage diagnosis of bridges.

## Figures and Tables

**Table 1:** Architecture of CNN with MPME.

No.	Layer	Function	Parameters
1	$F_{input}$	Input	L
2	$Conv_{1}$	1st Convolution	m×1×n
3	$P_{1}$	Pooling	*k*
4	$Conv_{2}$	2nd Convolution	m×1×n
5	$P_{2}$	Pooling	*k*
6	$Conv_{3}$	3rd Convolution	m×1×n
7	$P_{3}$	Pooling	*k*
8	$Conv_{4}$	4th Convolution	m×1×n
9	$P_{4}$	Pooling	*k*
10	$Conv_{5}$	5th Convolution	m×1×n
11	$P_{5}$	Pooling	*k*
12	MPME	Multi-channel Parallel Multi-scale Extractor	/
13	FC	Concat and Flatten	/
14	$F_{outp}$	Softmax	/

**Table 2:** Damage mode classification of Kando Bridge.

Damage Mode	Type	Degree	Load
N	No damage	No damage	20 kmph, car
$D_{1,1}$	Type I	case 1	20 kmph, car
$D_{1,2}$	Type I	case 2	20 kmph, car
$D_{2,1}$	Type II	case 1	20 kmph, car
$D_{2,2}$	Type II	case 2	20 kmph, car
$D_{3,1}$	Type III	case 1	20 kmph, car
$D_{3,2}$	Type III	case 2	20 kmph, car

**Table 3:** Damage condition of simulation model.

Condition	Unit	Degree (Percent)
N	No damage	No damage
$D_{1,1}$	Reduce the intensity of the portion near A2 in unit 6	10
$D_{1,2}$	Reduce the intensity of the portion near A2 in unit 6	20
$D_{2,1}$	12	5
$D_{2,2}$	12	15
$D_{3,1}$	16, 17, 18	15, 35, 5
$D_{3,2}$	16, 17, 18	15, 65, 5

**Table 4:** Damage mode classification of Seiran Bridge.

Mode	Type	Load
N	No damage	40 kmph, car
$D_{1,1}$	Bearing corrosion: case 1	40 kmph, car
$D_{1,2}$	Bearing corrosion: case 2	40 kmph, car
$D_{2,1}$	Steel frame corrosion: case 1	40 kmph, car
$D_{2,2}$	Steel frame corrosion: case 2	40 kmph, car
$D_{3,1}$	Concrete deck deterioration: case 1	40 kmph, car
$D_{3,2}$	Concrete deck deterioration: case 2	40 kmph, car

**Table 5:** Open-set transfer tasks.

Transfer Tasks	Source States	Training Data-Set	Testing Data-Set
$A_{1,1}:A\to B$	all	100% Labeled data-set A and 50% unlabeled data-set B	50% unlabeled data-set B
$A_{1,2}:A\to B$	$N,D_{1,1},D_{1,2},D_{2,1},D_{2,2},D_{3,1}$	100% Labeled data-set A and 50% unlabeled data-set B	50% unlabeled data-set B
$A_{2,1}:A\to C$	$N,D_{1,1},D_{1,2},D_{2,1},D_{3,1}$	100% Labeled data-set A and 50% unlabeled data-set C	50% unlabeled data-set C
$A_{2,2}:A\to C$	$N,D_{1,1},D_{1,2},D_{2,1}$	100% Labeled data-set A and 50% unlabeled data-set C	50% unlabeled data-set C
$B_{1,1}:B\to C$	$N,D_{1,2},D_{2,1},D_{3,1},D_{3,2}$	100% Labeled data-set B and 50% unlabeled data-set C	50% unlabeled data-set C
$B_{1,2}:B\to C$	$N,D_{1,2},D_{2,1},D_{2,2}$	100% Labeled data-set B and 50% unlabeled data-set C	50% unlabeled data-set C
$B_{2}:B\to A$	$N,D_{1,1},D_{1,2},D_{2,2},D_{3,1}$	100% Labeled data-set B and 50% unlabeled data-set A	50% unlabeled data-set A
$C_{1}:C\to A$	$N,D_{1,1},D_{1,2},D_{2,1},D_{3,1}$	100% Labeled data-set C and 50% unlabeled data-set A	50% unlabeled data-set A
$C_{2}:C\to B$	$N,D_{1,2},D_{2,1},D_{2,2},D_{3,1},D_{3,2}$	100% Labeled data-set C and 50% unlabeled data-set B	50% unlabeled data-set B

**Table 6:** Various transfer learning method.

Method No.	Method Name	Feature	Transfer Leaning Type
M1	CNN	Learned feature	No transfer
M2	OSVM	Learned feature	No transfer
M3	OSVM-MMD	Learned feature	MMD with OSVM
M4	DDC	Learned feature	MMD
M5	DCTLN	Learned feature	MMD with adversarial learning
M6	AWSDN	Learned feature	MCMK-WLMMD and adversarial auxiliary weighting

**Table 7:** Results of AWSDN in transfer B→A.

Condition	Precision (%)	Recall	Sample
N	98.52	1.00	200
$D_{1,1}$	92.75	0.92	200
$D_{1,2}$	90.12	0.91	200
$D_{2,2}$	92.91	0.92	200
$D_{3,1}$	95.65	0.97	200
Unknown	95.08	0.93	400
Average	94.17	0.94	
